# Effects of unilateral decompressive craniectomy on patients with unilateral acute post-traumatic brain swelling after severe traumatic brain injury

**DOI:** 10.1186/cc8178

**Published:** 2009-11-23

**Authors:** Wusi Qiu, Chenchen Guo, Hong Shen, Keyong Chen, Liang Wen, Hongjie Huang, Min Ding, Li Sun, Qizhou Jiang, Weiming Wang

**Affiliations:** 1 Department of Neurosurgery, Hangzhou Second Hospital, College of Medicine, Hangzhou Normal University, 126 Wenzhou Road, Hangzhou, 310015, China; 2 Brain Medicine Research Institute, College of Medicine, Zhejiang University, 88 Jiefang Road, Hangzhou, 310009, China; 3 Department of Neurosurgery, Second Affiliated Hospital, College of Medicine, Zhejiang University, 88 Jiefang Road, Hangzhou, 310009, China

## Abstract

**Introduction:**

Acute post-traumatic brain swelling (BS) is one of the pathological forms that need emergent treatment following traumatic brain injury. There is controversy about the effects of craniotomy on acute post-traumatic BS. The aim of the present clinical study was to assess the efficacy of unilateral decompressive craniectomy (DC) or unilateral routine temporoparietal craniectomy on patients with unilateral acute post-traumatic BS.

**Methods:**

Seventy-four patients of unilateral acute post-traumatic BS with midline shifting more than 5 mm were divided randomly into two groups: unilateral DC group (n = 37) and unilateral routine temporoparietal craniectomy group (control group, n = 37). The vital signs, the intracranial pressure (ICP), the Glasgow outcome scale (GOS), the mortality rate and the complications were prospectively analysed.

**Results:**

The mean ICP values of patients in the unilateral DC group at hour 24, hour 48, hour 72 and hour 96 after injury were much lower than those of the control group (15.19 +/- 2.18 mmHg, 16.53 +/- 1.53 mmHg, 15.98 +/- 2.24 mmHg and 13.518 +/- 2.33 mmHg versus 19.95 +/- 2.24 mmHg, 18.32 +/- 1.77 mmHg, 21.05 +/- 2.23 mmHg and 17.68 +/- 1.40 mmHg, respectively). The mortality rates at 1 month after treatment were 27% in the unilateral DC group and 57% in the control group (p = 0.010). Good neurological outcome (GOS Score of 4 to 5) rates 1 year after injury for the groups were 56.8% and 32.4%, respectively (p = 0.035). The incidences of delayed intracranial hematoma and subdural effusion were 21.6% and 10.8% versus 5.4% and 0, respectively (p = 0.041 and 0.040).

**Conclusions:**

Our data suggest that unilateral DC has superiority in lowering ICP, reducing the mortality rate and improving neurological outcomes over unilateral routine temporoparietal craniectomy. However, it increases the incidence of delayed intracranial hematomas and subdural effusion, some of which need secondary surgical intervention. These results provide information important for further large and multicenter clinical trials on the effects of DC in patients with acute post-traumatic BS.

**Trial registration:**

ISRCTN14110527

## Introduction

Acute post-traumatic brain swelling (BS), one variety of the pathological forms of diffuse brain injury, has been described as the increase of brain size in the several hours following traumatic brain injury (TBI) without obvious intracranial hematoma [1-7]. The pathophysiological entity of post-traumatic BS is not yet fully understood [5,7]. It has been indicated that cytotoxic edema, rather than vasogenic edema or cerebral vasocongestion, could contribute to this phenomenon. Acute post-traumatic BS can be classified into hemisphere BS and diffuse BS based on computed tomography (CT) scans [2,5,7-9]. Without appropriate management, the subsequent increase of intracranial pressure (ICP) with extensive swelling and distension of cerebral tissues would lead to the deterioration of patients with TBI in the critical care department [1-4]. If the initial medical management, such as sedation, drainage of cerebrospinal fluid (CSF), and osmotherapy, is refractory to the intracranial hypertension, the second tier of management such as therapeutic hypothermia management or decompressive craniectomy (DC) should be considered [2,5,6].

Despite maximal medical management, such as dehydration and pentobarbital coma, there is still a high risk of morbidity and death in patients with traumatic BS [1-3,5,7]. It has also been indicated that surgical decompression should be routinely performed before irreversible brain damage occurs for some subgroups of patients with post-traumatic BS. The rationale for decompression is to reduce ICP, to prevent herniation and the vicious circle of further BS, and to combat the deleterious effects of TBI. Previous studies, including ours, have demonstrated that acute post-traumatic BS should be diagnosed correctly and promptly with CT scanning as soon as possible, and for patients with midline shift of more than 5 mm, especially with thin-layered subdural hematoma, surgical intervention is essential to reduce the fatality of acute post-traumatic BS [5,7-10].

Although the importance of DC in the treatment of traumatic BS has been demonstrated in several studies, there are rare results from randomized trials to confirm or refute the effectiveness of DC in adults, and the criterion and the safety of this treatment has not been fully recognized [5,7-10]. DC for some subgroups of patients with acute post-traumatic BS may be useful, because this kind of treatment may be a useful option when maximal medical treatment has failed to control refractory high ICP in TBI resulting from the acute post-traumatic BS [4,9,11,12].

To further investigate the therapeutic effects and the complications of unilateral DC on post-traumatic BS, we conducted a clinical trial, and the effects as well as the main complications were analyzed.

## Materials and methods

### Study design

This clinical study was designed as a prospective randomized clinical trial. The research protocol was approved by the Institutional Review Board and the ethical committees of Clinical Medical College of Hangzhou, according to Good Clinical Practice standards, and Declaration of Helsinki principles were strictly followed. As the patients in this study were incapable of granting informed consent, investigators therefore obtained informed consent from the subject's legal guardian or healthy proxy before accepting the advised treatment.

After informed consent was obtained from the patient's family, the patient was assigned to one of the following two groups using a randomization table. Allocation and randomization was concealed and the investigators were not aware to which group the patient would be assigned, and the allocation sequence was protected until assignment. The physicians in charge of the patient were not involved in data collection, and the nursing staff and the surgical team were not aware of the patient's group assignment. A single trained assessor and the data analyzer were blind to the treatment group. Therefore, biased grouping was avoided, and adherence to the principles of control, random, equilibrium was ensured.

### Patient population and grouping

Of 7291 adult in-patients with severe TBI admitted to our department between 2000 and 2008, 74 patients with acute post-traumatic BS with midline shifting more than 5 mm were enrolled in our clinical trial. There were 51 male and 23 female patients with severe TBI, with age ranging from 18 to 65 (40.1 ± 11.8) years. All the patients met the following criteria: a history of TBI, Glasgow Coma Scale (GCS) of 8 or less at admission, and swollen hemisphere (43 left and 31 right, with midline shift >5 mm and contusions <25 ml and compressed basal cisterns) apparent on CT scans. There were 10 cases of traumatic cerebral hemisphere infarction, 13 cases with a thin layer of subdural hematoma (less than 2 mm, and not consistent with the midline shift), 19 cases with midline shift more than 10 mm, and 51 cases with compressed or obliterated basilar cistern and/or cisterna ambiens. Sequential CT confirmed the diagnosis within 1 to 19 hours (mean 5.1 hours).

Patients below the age of 18 years or above 65 years, multiply-injured patients, those with any previous disabling neurological disease, intracerebral haematoma of more than 3 cm in diameter, previous craniectomy, extra-axial haematoma greater than 0.5 cm in thickness, spinal cord injury, penetrating brain injury, fixed dilated pupils and GCS score of 3 with no chance of survival, were excluded.

Craniotomy was undergone for all patients from 2 to 24 hours (mean 5.8 hours) after admission, and randomized into two groups as follows: unilateral DC group (n = 37) and unilateral routine temporoparietal craniectomy group as control group (n = 37). The main characteristics of the patients are shown in Table 1.

**Table 1:** Main clinical and demographic characteristics between the two groups

Variable	Unilatereal DC group (n = 37)	Control group (n = 37)	*P-values*
Number of male patients (%)	27 (73.0)	24 (64.9)	0.616
Mean age (years)	39.9 ± 1.9	40.2 ± 11.9	0.895
GCS score			
3-5 (%)	9 (24.3)	10 (27.0)	0.79
6-8 (%)	28 (75.7)	27 (73.0)	0.79
Mechanism of injury			
Motor vehicle (%)	30 (81.1)	29 (78.4)	0.772
Fall (%)	5 (13.5)	7 (18.9)	0.528
Other (%)	2 (5.4)	1 (2.7)	0.556
Marshall CT scan classification			
I (%)	0	0	
II (%)	0	0	
III (%)	5 (16.2)	3 (8.1)	0.454
IV (%)	16 (43.2)	17 (45.9)	0.815
V (%)	14 (37.8)	14 (37.8)	1
VI (%)	2 (5.4)	3 (8.1)	0.643

CT = computed tomography; DC = decompressive craniectomy; GCS = Glasgow Coma Scale.

### Management procedures

Based on the guidelines for the management of severe head injury set forth by the Joint Section on Neurotrauma and Critical Care of the Brain Trauma Foundation and the American Association of Neurological Surgeons, all patients underwent lateral craniotomy within 24 hours after injury and other medical management such as dehydration with mannitol (average amount received in subsequent six-hour period was 125 ml 20% mannitol) and routine pharmacological or physical measures adopted to maintain normal body temperature after diagnosis of post-traumatic BS accordingly.

The surgery mode of DC was elective at the frontoparietotemporal region, based on the lesion location and midline shift determined by CT scans [7]. Briefly, the bone window was about 15 cm in diameter with duraplasty using expanded dura substitute when necessary. The anterior was frontal to the midpupillary lines, and the posterior line was about 3 to 4 cm posterior to the external acustic meatus. The superior line was 2 cm of the lateral edge of the superior sagittal sinus, and the inferior line was extended below the level of the zygomatic arch, so that the frontal and temporal base could be explored. Durotomy was performed over the entire region of bony decompression as a stellate shape. Cranioplasty was performed after three months.

Postoperative therapy was similar to that previously described, which included elevation of the upper part of the body by 15 to 30 degrees, keeping the respiratory tract unobstructed (with assisted ventilation to ensure oxygen saturation of 95% or more against hypoxia and partial pressure of arterial carbon dioxide maintained at about 40 mmHg at 37°C), maintaining intubation, sedation and continuous muscle relaxation if necessary, controlling the blood glucose level and body temperature and balancing water and electrolyte levels, preventing dehydration and diuresis, maintaining homeostasis balance and anti-infection with sensitive antibiotics, ensuring normothermia and so on [5,7-10].

The control group received the unilateral routine temporoparietal craniectomy and the operation site depended on the location of injury and midline shift accordingly [7]. The bone window diameter was about 8 cm and the dura mater was also fixed at the edge with duraplasty, and cranioplasty was also performed after three months. In the routine craniotomy group, postoperative treatment was similar to those in the unilateral DC group.

### Monitoring parameters

The following parameters of the patients were monitored at admission and after craniotomy. (1) The temperature, heart rate, respiration rate and blood pressure, arterial oxygen saturation with the multiple monitor (Model NO: 90309, Space Lab, Medical. Inc., Issaquah, Washington, USA). The data were recorded at every 12 hours for 7 days after craniotomy. (2) Continuous recording of ICP was applied in all patients for 96 hours with the ICP monitor system (Camino-MPM1, Integra LifeSciences CO, Plainsboro, New Jersey, USA.). A cranial hole was drilled, if necessary, and the drainage catheter was induced 5 cm or so into the right lateral ventricles via the level of the anterior horn. The intraventricular pressure probe (Camino-110-4B Integra LifeSciences CO, Plainsboro, New Jersey, USA) was placed at the level of foramen of Monro during craniotomy. (3) Complications. Mainly inclusive of delayed intracranial hematoma, pulmonary infection, digestive tract hemorrhage, and electrolytes disorders (beyond the normal serum concentrations of Na^+^, Cl^-^, Mg^2+^, K^+^, P^3+ ^and Ca^2+^). The data were recorded every 12 hours for 7 days, and every 24 hours for another 7 days after craniotomy. (4) Glasgow Outcome Scale (GOS) scores, from 1 to 5 respectively, according to: death, vegetative state, severe disability, moderate disability, mild or no disability, evaluated at one year follow-up after injury.

### Statistical analysis

The ICP levels are presented as mean ± standard deviation, and were analyzed with one-way analysis of variance. Neurological outcomes were evaluated by Cochran-Mantel-Haenszel test and by chi-squared tests. Data were measured blind to treatment group, and were analyzed using SAS statistical software (version 8; SAS Institute, Cary, NC, USA). A probability value less than 0.05 was defined as significant difference.

## Results

### The vital signs

The temperature, heart rate, respiration rate and blood pressure were kept at normal levels with above-mentioned appropriate treatment at every 12 hours for 7 days after craniotomy. There was no significant difference of abnormality of vital signs between the two groups.

### ICP values

The mean ICP values of patients in the unilateral DC group at 24, 48, 72 and 96 hours after injury were significantly lower (about 30%) than those of the routine temporoparietal craniectomy group (15.19 ± 2.18 mmHg, 16.53 ± 1.53 mmHg, 15.98 ± 2.24 mmHg and 13.518 ± 2.33 mmHg versus 19.95 ± 2.24 mmHg, 18.32 ± 1.77 mmHg, 21.05 ± 2.23 mmHg and 17.68 ± 1.40 mmHg, respectively). Accordingly, the highest ICP was observed 72 or 48 hours after injury in the groups, which is consistent with the previous studies.

### Neurological outcomes

The mortality rates one month after craniotomy were 27% in the unilateral DC group as compared with 57% in control group (*P *= 0.010). According to the GOS scores one year after injury, significant difference in overall neurological outcomes between both groups was found. The difference of good neurological recovery (GOS score 4 to 5) between the unilateral DC group and control group was significant (56.8% versus 32.4%, Table 2).

**Table 2:** Clinical outcome of two groups at six months follow-up (n, (%))

GOS scores	Unilatereal DC group (n = 37)	Control group (n = 37)
5	15 (41)	5 (14)
4	6 (16)	7 (19)
3	5 (14)	4 (11)
2	1 (3)	0
1	10 (27)	21 (57)

DC = decompressive craniectomy; GOS = Glasgow Outcome Scale.

### Complications

There was no evidence of severe complications related to DC. There were two cases of cenencephalocele (5.4%) in the unilateral DC group. With the bulged brain tissue removed, one case died in one week and the other remained in a vegetative state. Delayed intracranial hematoma was seen in eight cases and contralateral subdural effusion in two cases. Evacuation of hematoma with secondary craniotomy was undergone for four cases, and conservative treatment and subdural effusion-peritoneal-shunt for every two cases. Therefore, there were four cases of subdural effusion (10.8%) for patients in the DC group. There were six cases of cenencephalocele (16.2%) in the control group. With bone window enlarged and the cenencephalocele brain tissue removed, four cases died of central respiratory failure within three days, and the remaining two cases died within 10 days.

There were two cases of subdural hematoma (5.4%) in the control group, and they were cured with conservative treatment. There was one case of intracranial infection confirmed by CSF germiculture, but it was cured with recurrent lumbar puncture, CSF drainage and other anti-infection measures. The incidence rate of pulmonary infection, hemorrhage in digestive tract, hypoglycemia (blood sugar <2.6 mmol/L), blood cell counts, electrolyte disorders or renal malfunction in two groups was not significant (Table 3).

**Table 3:** Main complications of patients with unilateral brain swelling after severe traumatic brain injury

Variable	Unilatereal DC group (n = 37)	Control group (n = 37)	*P-values*
Cenencephalocele (%)	2 (5.4%)	6 (16.2%)	0.134
Delayed intracranial hematoma (%)	8 (21.6%)	2 (5.4%)	0.041
Subdural effusion (%)	4 (10.8%)	0	0.040
Intracranial infection (%)	1 (2.7)	0	0.314
Pulmonary infection (%)	11 (29.7)	13 (35.1)	0.619
Hemorrhage in digestive tract (%)	17 (45.9)	20 (54.0)	0.485
Hypoglycemia (%)	12 (32.4)	13 (35.1)	0.806
Thrombocytopenia (%)	8 (21.6)	7 (18.9)	0.772
Electrolyte disorders (%)	19 (51.4)	23 (62.2)	0.348
Renal malfunction (%)	13 (35.3)	9 (24.3)	0.309

DC = decompressive craniectomy

As shown above, the incidences of delayed intracranial hematoma and subdural effusion were higher in the unilateral DC than in the control group (21.6% and 10.8% versus 5.4% and 0, respectively, *P *= 0.041 and 0.040). All the complications were treated and improved without severe sequelae. There were no severe abnormalities of heart rate, respiration rate, and blood pressure and arterial blood gas analysis with above-mentioned appropriate treatment.

## Discussion

Although BS could be alleviated by mannitol infusion even without craniotomy, a subset of patients with malignant BS after TBI could not be easily manageable, and diffuse unilateral or bilateral BS has definitive correlation with clinical deterioration with brain herniation [5,13-17]. The use of DC for severe TBI was rekindled with the demonstration that DC improved the outcome in the studies of ischemic and traumatic injury. Our study also confirmed that unilateral DC had positive effects on acute post-traumatic BS with a midline shift of more than 5 mm. Furthermore, DC seems also to be a successful option in the treatment of diffuse brain edema after subarachnoid haemorrhage or space occupying infarction due to vasospasm after subarachnoid haemorrhage.

It is very difficult to decide what kind of operation can not only reduce ICP but also protect the cerebral tissue from secondary brain injury resulting from hypoperfusion and/or hypopressure [9,10,18]. Although DC has been rekindled in different studies, this procedure is neither first-line treatment nor standard of care in severe TBI. The time of the DC is one important factor, especially for post-traumatic BS with subdural hematoma. Our study demonstrated that acute post-traumatic BS could coexist with subdural hematoma, and unilateral DC has superiority in lowering ICP, reducing the mortality rate and improving neurological outcomes over routine temporoparietal craniectomy. This is consistent with previous studies [5,8,12,13,16].

DC can reduce the incidence of cenencephalocele, although an increase is seen in the incidence of some complications such as intracerebral hematoma, hygromas and hydrocephalus. However, it seemed that the complication rate after DC was low and has no serious influence on the patient's prognosis [5,10,16]. In the present study, the early complications including upper gastrointestinal hemorrhage and hypoglycemia are not specific to DC and can be treated and improve without severe sequelae, but the late complication such as subdural effusion is a critical one. Our results strongly suggest that even with the favorable neurological outcome, unilateral DC increases the incidence of delayed intracranial hematomas and subdural effusion, some of which need further surgical intervention. It could largely be seen as a last resort therapeutic option. Routine DC should not be recommended for all patients with post-traumatic BS [1,5,7,9,13,15].

Although DC is being increasingly investigated for brain injury irrespective of the underlying disease, the effects of this procedure on clinical outcome are still controversial [1,2,5,7,18-23]. We agree on the idea that the application of DC in severe TBI lacks enough class I evidence relevant to this topic, and this study aims to conduct the prospective trials on acute post-traumatic BS. To our knowledge, the following factors are critical in surgical management of post-traumatic BS: (1) The trauma type, as well as the criteria in deciding the type of BS after TBI is important [2,5,13]. Decompression is not imperative for all patients with traumatic BS. For BS without obvious midline shift, it might progressively resolve and the clinical state could improve with appropriate treatment such as dehydration. (2) The time, type and techniques of DC should also be taken into account in perioperative treatment [2,5,10,13,15,19]. If DC can be accomplished before the ICP value exceeds 40 mmHg for a sustained period and within 48 hours of the time of injury, the potential to influence outcome is greatest. As thin-layed subdural hematoma may be associated with severe neurological prognosis, and elevated ICP is one of the major deteriorating factors in patients with TBI, the prevention of intracranial hypertension by decompression plays a key role with respect to secondary brain injury. Therefore, if conventional therapy fails in patients suffering from decompensated intracranial hypertension, the existence of thin-layed subdural hematoma with BS after TBI may need decompression as soon as possible, or within 48 hours. In our study, patients underwent DC within 24 hours, and all patient outcomes were favorable. The decision to use bilateral or unilateral surgery must be based on the neurological and radiological findings of each patient [5,7,15,19]. (3) Prophylactic measurement of the possible complications after DC. Besides the duraplasty and other treatment for prevention of CSF leakage, the technique and therapeutic measures against side effects after DC are essential. Some would develop contralateral or bilateral subdural hematoma and/or subdural effusion, which may be attributable to DC [9,10,20,21].

The studies reporting complications of DC are rare, some of which are subdural effusion [12,15,21-28]. When the occupying effects of subdural hematoma/effusion are present, effective treatment should be applied. Although the definitive management is burr-hole drainage of the fluid collection, no consensus exists on which patients may benefit from surgical intervention [2,5,12,18,23]. Other treatment interventions such as hyperventilation, or the use of mannitol, steroids, or phenobarbitol have been proposed as adjuncts to management of patients with TBI. However, internal drainage is the last resort for the symptomatic subdural effusion (especially with the mass effect) after DC described here. The supportive treatment such as effective monitoring of ICP, anti-infection and basic nursing inclusive of exercising limbs in order to prevent phlebothrombosis is imperative. As early craniotomy put another impact on the patient, the mean arterial pressure should be kept within normal limits and hypoxia should be avoided [24-30].

## Conclusions

Although the application of DC in severe TBI is controversial and the population in the present study is small, our study demonstrated that unilateral DC had superiority in lowering ICP, reducing the mortality rate and improving neurological outcomes over routine temporoparietal craniectomy [10,25,26]. Furthermore, the indication for DC should be individualized. However, our study has demonstrated that unilateral DC increased the incidence of delayed intracranial hematomas and subdural effusion, some of which need further surgical intervention. The present results strongly support further large, multicenter clinical trials to evaluate the difference of the effects between DC and other means of craniotomy with proper evaluation, or of the effects of a combination with other methods such as hypothermia, of the therapies in acute post-traumatic BS after severe TBI [10,24-26,31-34].

## Key messages

• Acute post-traumatic BS needs emergent treatment following traumatic brain injury, and there is controversy about the effects of craniotomy on acute post-traumatic BS.

• Our data suggest that unilateral decompressive craniectomy is superior to routine temporoparietal craniectomy on patients with unilateral acute post-traumatic BS after severe TBI, but it increases the incidence of delayed intracranial hematoma and subdural effusion, some of which need secondary surgical intervention.

## Abbreviations

BS: brain swelling; CSF: cerebrospinal fluid; CT: computed tomography; DC: decompressive craniectomy; GCS: Glasgow Coma Scale; GOS: Glasgow Outcome Scale; ICP: intracranial pressure; TBI: traumatic brain injury.

## Competing interests

The authors declare that they have no competing interests.

## Authors' contributions

WQ and HS participated in the trial design and were involved in the study analysis and summary. WQ, CG, QJ and WW obtained the data. LW, KC, HH, MD and LS participated in the data analysis and interpretation of the results. All authors reviewed the final version.
